# Uterine Insulin Sensitivity Defects Induced Embryo Implantation Loss Associated with Mitochondrial Dysfunction-Triggered Oxidative Stress

**DOI:** 10.1155/2021/6655685

**Published:** 2021-04-12

**Authors:** Meixia Chen, Jie Li, Bo Zhang, Xiangfang Zeng, Xiangzhou Zeng, Shuang Cai, Qianhong Ye, Guangxin Yang, Changchuan Ye, Lijun Shang, Shiyan Qiao

**Affiliations:** ^1^State Key Laboratory of Animal Nutrition, China Agricultural University, Beijing 100193, China; ^2^Beijing Biofeed Additive Key Laboratory, China Agricultural University, Beijing 100193, China

## Abstract

**Methods and Results:**

Herein, a comprehensive proteomic analysis was conducted on proliferative endometria from sows with low and normal reproductive performance (LRP and NRP, respectively). Enrichment analysis of differentially expressed proteins revealed alterations in endometrial remodeling, substance metabolism (mainly lipid, nitrogen, and retinol metabolism), immunological modulation, and insulin signaling in LRP sows. Importantly, aberrant lipid metabolite accumulation and dysregulation of insulin signaling were coincidently confirmed in endometria of LPR sows, proving an impaired insulin sensitivity. Furthermore, established high-fat diet- (HFD-) induced insulin-resistant mouse models revealed that uterine insulin resistance beginning before pregnancy deteriorated uterine receptivity and decreased implantation sites and fetal numbers. Mitochondrial biogenesis and fusion were decreased, and reactive oxygen species was overproduced in uteri from the HFD group during the implantation period. Ishikawa and JAR cells directly demonstrated that oxidative stress compromised implantation *in vitro*.

**Conclusions:**

This study demonstrated that uterine insulin sensitivity impairment beginning before pregnancy resulted in implantation and fetal loss associated with oxidative stress induced by mitochondrial dysfunction.

## 1. Introduction

Early pregnancy loss contributes greatly to declined fertility rates in humans and mammalian animals [1]. In humans, about 15% of pregnancies result in pregnancy loss [2], and 40% to 50% of pregnant women failed to develop pregnancy beyond 20 weeks, among which, 75% of this failure is caused by implantation loss during early pregnancy [3, 4]. Among the mammalian animals, pregnancy loss for pigs is the highest, accounting for about 20% to 45% [5], and 20% to 30% of this loss occurs during the peri-implantation period [6]. Therefore, alleviating implantation losses is of importance to improve pregnancy outcomes for humans and mammalian animals.

Implantation loss is influenced by oocyte quality [7], ovarian development [8], and uterine function [9]. The endometrium, which provides the site for embryo implantation and development, undergoes fine-tuned morphological and physiological changes in a cycle-dependent manner. Embryos only successfully implant in the endometrium during a short period in the midsecretory stage. Many researches have studied the secretory endometrium [10–14], but only a few are known about the proliferative endometrium. The endometrium during the proliferative phase mainly undergoes cellular proliferation and extracellular matrix remodeling, which is fundamentally important for subsequent embryo implantation during the secretory phase [15, 16]. Bergeron et al. found some endometrial maturation disturbances during the secretory phase can be traced to disorders during the proliferative phase [17]. Aldad et al. reported that endometrial growth deficiency during the proliferative phase may be a common abnormality in women with infertility and repeated pregnancy loss [18]. However, the underlying mechanisms causing proliferative endometrial defects which elicit implantation loss have not been fully studied.

Pigs present an excellent animal model for studying human nutrition and reproductive physiology, as they share high similarity to humans in terms of pathological, physiological, and anatomical features [19]. Therefore, to dissect the underlying mechanisms of proliferative endometrial defects in implantation loss, a comprehensive proteomic analysis of proliferative endometria from low and normal reproductive sows was conducted. Here, we identified disorders in remodeling, substance metabolism, and immunological modulation and compromised uterine local insulin sensitivity in endometria of low reproductive sows. Results from the high-fat diet-induced insulin-resistant mouse model and Ishikawa cells revealed that uterine insulin resistance beginning before the pregnancy deteriorates uterine function through mitochondrial dysfunction causing oxidative stress, resulting in compromised implantation sites and fetal numbers. Our findings shed lights on the understanding of the etiology of the uterine dysfunction-driven reproductive disorders and provided molecular mechanisms behind uterine dysfunction under the uterine insulin-resistant state, highlighting the importance of preventing/reverting treatment before pregnancy.

## 2. Materials and Methods

### 2.1. Ethics Statement

Animal handling procedures were approved by the Institutional Animal Care and Use Committee of China Agricultural University (ID: SKLAB-B-2010-003).

### 2.2. Sow Housing and Endometrial Collection

Healthy Landrace × Large White sows (*n* = 10) with similar parity and different reproductive performance were used: normal reproductive performance (NRP) sows (*n* = 5, average live litter size over the last three parities = 12.53 ± 0.46) and low reproductive performance (LRP) sows (*n* = 5, average live litter size over the last three parity = 8.07 ± 0.52) [20]. Sows had free access to drinking water and diets that met the nutrient requirements of swine (2012) [21]. In the preovulatory follicular phase, sows were killed by electric shock. Blood samples were collected and handled as described by Chen et al. [20], and intact proliferative endometrial tissues were immediately separated [22]. All samples were stored in -80°C until analysis.

### 2.3. Proteomic Sample Preparation and Label-Free Proteomic Analysis of Endometrial Tissues

Proteomic samples were prepared using endometria from NRP and LRP sows [23]. Label-free analysis of peptides was achieved on an Eksigent 425 (AB SCIEX) LC system coupled with a Q-Exactive mass spectrometer (Thermo Scientific, Bremen, Germany). Sequence database searching and protein identification were conducted. The false discovery rate (FDR) was strictly limited to <0.01. The NRP group was set as the control to the LRP group. Proteins with a fold change (FC) > 1.5 (or <0.6667) and *p* < 0.05 were regarded as differentially expressed proteins (DEPs). Detailed methods are described in Supporting Information.

### 2.4. Bioinformatics Analysis and Pathway Analysis

Molecular functions, biological processes, and cellular components of DEPs were analyzed by gene ontology (GO) enrichments. ReviGO (http://revigo.irb.hr/) was employed for deducing redundancy of enriched GO terms. Cytoscape (version 3.5.1) was used to visualize and summarize GO enrichment results. Kyoto Encyclopedia of Genes and Genomes (KEGG) enrichment was also conducted based on all DEPs. The threshold of significant GO terms and KEGG pathways was set at *p* < 0.05.

### 2.5. Mouse Housing

Seven-to-eight-week-old female ICR mice (Charles River Laboratories, China Inc.) were kept under controlled conditions (12 h light and 12 h night, 22°C-25°C). Mice were randomly assigned to two groups: feeding a normal diet (D1032, Beijing HFK Bioscience Co. Ltd.) or a high-fat diet (HFD, D12492, Research Diet, Inc.). After 16 weeks, mice were mated with normal male mice. Pregnant mice were continued with a control diet or HFD. Uteri (implantation sites) and serum were collected, and implantation site/fetal number was recorded.

### 2.6. Glucose Tolerance Tests and Insulin Tolerance Tests

After 16 weeks, six to nine mice per diet were randomly selected for glucose tolerance test (GTT) and insulin tolerance test (ITT) [24]. For GTT, mice were fasted overnight and then intraperitoneally injected with glucose (2 g per kg body weight). For ITT, mice were intraperitoneally injected with insulin (0.75 IU per kg body weight). Blood samples were harvested from the tail vein at 0, 30, 60, 90, and 120 min after glucose or insulin administration. Glucose concentrations were immediately measured using a HemoCue B glucose analyzer (Angelholm, Sweden).

### 2.7. Index Measurements in Serum and Endometrium

Concentrations of glucose, insulin, adenosine monophosphate (AMP), adenosine triphosphate (ATP), triglyceride (TG), leptin, malonyl-CoA, and diacylglycerol in the endometrium, as well as glucose, TG, low-density lipoprotein-cholesterol (LDL-C), high-density lipoprotein-cholesterol (HDL-C), cholesterol (CHOL), and insulin in serum, were determined using assay kits (Biosino Bio-Technology and Science Inc., Beijing, China) according to the manufacturer's instructions. Insulin resistance was estimated using the homeostatic model assessment method (HOMA-IR) [25], which was expressed as insulin (mIU/L) × glucose (mmol/L)/22.5.

### 2.8. Hormone Measurement

Concentrations of 17*β*-estradiol and progesterone in mouse serum were determined using enzyme-linked immunosorbent assay kits (Cusabio, Wuhan, China, http://www.cusabio.com/) according to the manufacturer's instructions.

### 2.9. Western Blotting

Total protein from sow endometria and mouse uteri was extracted as described above. Equal amounts of protein were loaded and separated on SDS-PAGE gels and then transferred to PVDF membranes (0.45 *μ*m, Millipore, MA, USA). After blocking with 5% skim milk or bovine serum albumin for 1 h at room temperature, membranes were incubated with corresponding primary antibodies (Supplemental Table 2) overnight at 4°C. After five washes with TBST, membranes were incubated with DyLight 800-labeled secondary antibodies (Cell Signaling Technology, MA, USA) for 2 h at room temperature. Membranes were washed and then visualized with the Odyssey CLx (LI-COR, NE). Band densities were quantified using the ImageJ software. *β*-Actin was used as the loading control.

### 2.10. RNA Preparation and Quantitative RT-PCR

Total RNA was isolated using RNAiso Plus reagent (9109, Takara Bio, Kusatsu, Japan), and cDNA was synthesized using the PrimeScript RT reagent Kit with gDNA Eraser (RR047A, Takara Bio, Kusatsu, Japan) according to the manufacturer's instructions. Quantitative RT-PCR was performed in a LightCycler 96 system (Roche Diagnostics, Basel, Switzerland) using TB Green® Premix Ex Taq™ II (Tli RNaseH Plus) (RR820A, Takara Bio, Kusatsu, Japan) following the manufacturer's instructions. *β*-Actin was used as an internal control. The primers used in this work are presented in Supplemental Table S3. Relative mRNA expression level was calculated using the 2^-*ΔΔ*t^ method [26].

### 2.11. Cell Culture

Ishikawa cells and JAR cells were cultured in the RMPI 1640 medium (Gibco, USA) supplemented with 10% fetal bovine serum (Gibco, Australia) in a humidified atmosphere with 5% CO_2_.

### 2.12. ROS Level Measurement

ROS levels in mouse uteri and Ishikawa cells were determined using the DCFH-FA kit (Nanjing Jiancheng, Nanjing, China) according to the manufacturer's instructions.

### 2.13. *In Vitro* Implantation Model

*In vitro* implantation was performed using Ishikawa and JAR cells as described previously with minor modification [27]. A detailed protocol is described in the Supporting Information.

### 2.14. Statistical Analysis

Results are expressed as the mean ± SEM. For results of index measurements in serum and endometria, qPCR, and western blot, data were analyzed by Student's *t* test using SAS software (version 9.0). For the results of the proteomics, *t* test, volcano plot, one-way ANOVA, partial least squares-discriminant analysis (PLS-DA) and post hoc analysis as well as correlation analysis were used. *p* < 0.05 was considered significant.

## 3. Results

### 3.1. Proteomic Profiling of the Endometria from NRP Different from LRP Sows

**Figure 1:**
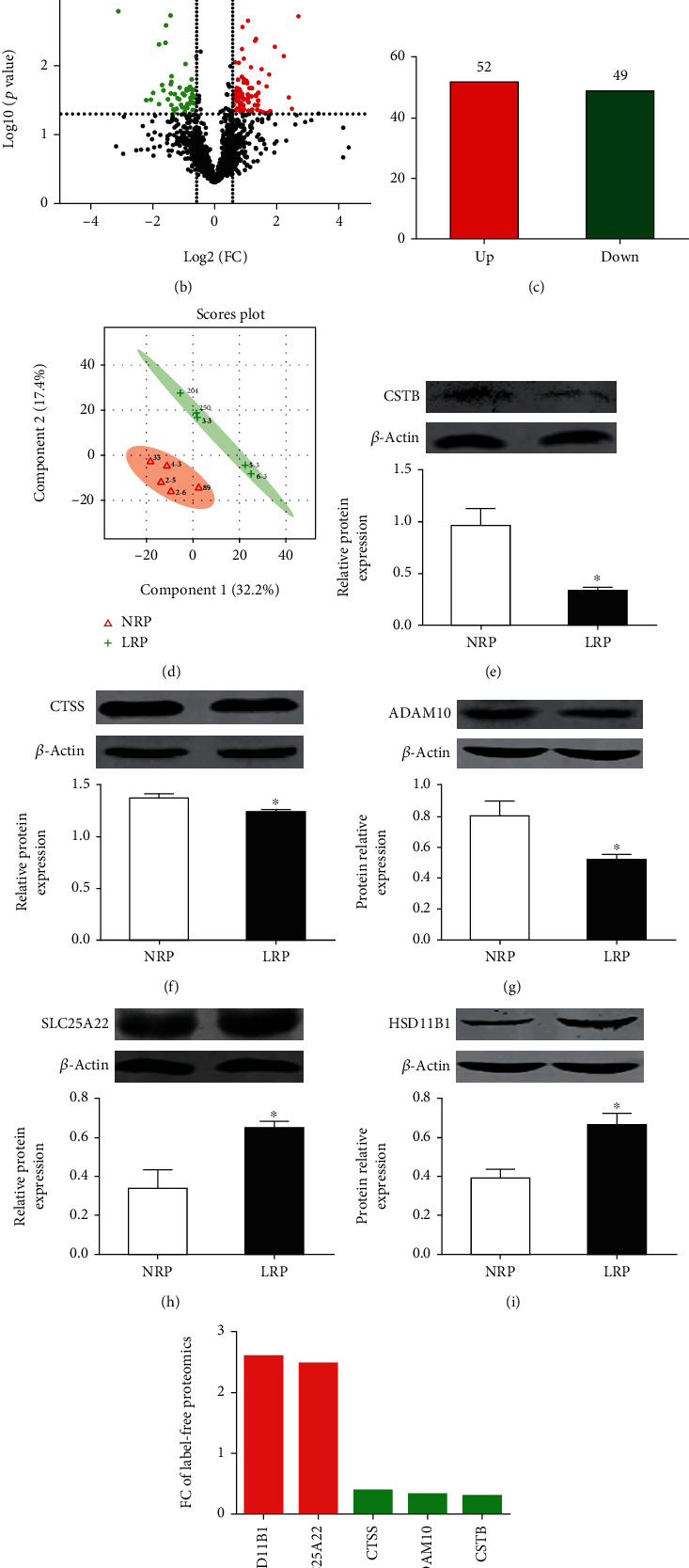
Proteomic profiling of endometria from NRP different from LRP sows. (a) Identification summary of label-free quantitative proteomic results. *n* = 5. (b) The scatter plot. Proteins with *p* < 0.05 and fold change ≥ 1.5 (or ≤0.67) were deemed as differentially expressed proteins (DEPs). Upregulated and downregulated DEPs were shown in red and green, respectively. (c) A total of 52 proteins were upregulated, and 49 were downregulated in the endometria of LRP sows. (d) Multivariate partial least squares-discriminant analysis (PLS-DA) scatted plots clearly distinguished LRP endometria from NRP endometria. (e–i) Relative protein levels of CSTB, CTSS, ADAM10, SLC25A22, and HSD11B1 were assessed by western blot (*n* = 3). *β*-Actin was used as a loading control. (j) Fold change of those proteins quantified in label-free proteomic data. LRP: low reproductive performance; NRP: normal reproductive performance. Fold change, LRP sows relevant to NRP sows. Data are expressed as mean ± SEM. Student's *t* test was used for statistical analysis. ^∗^*p* < 0.05.

To explore the underlying mechanisms of proliferative endometrial defects implicated in implantation failure/loss, a label-free quantitative proteomic analysis was performed using the proliferative endometria derived from LRP and NRP sows. The experimental flowchart is shown in Figure S1. SDS-PAGE bands and peptide length distribution demonstrated the high reliability of sample preparation for proteomic analysis (Figure S2). Based on at least two unique peptides and the criteria of FDR < 0.01 on both peptide and protein level, 148,268 peptides and 3,318 unique proteins were identified (Figure 1). Among them, 101 DEPs with *p* < 0.05 and FC ≥ 1.50 (or FC ≤ 0.67) were identified. There were 52 upregulated proteins (red) and 49 downregulated proteins (green) (Figure 1(b)). Next, expressions of five randomly selected proteins, including cystatin-B (CSTB), cathepsin S (CTSS), disintegrin and metalloproteinase domain-containing protein 10 (ADAM10), mitochondrial glutamate carrier 1 isoform X1 (SLC25A22), and corticosteroid 11-beta-dehydrogenase isozyme 1 (HSD11B1) were determined using western blot. As shown in Figures 1(e)–1(j), high consistence between western blot and proteomic data indicated the high reliability of our label-free proteomic data. Based on PLS-DA analysis, LRP endometria could be easily distinguished from NRP endometria (Figure 1(d)) indicating there may be a distinct regulatory network in NRP and LRP endometria.

### 3.2. GO Enrichment Analysis of DEPs

**Figure 2:**
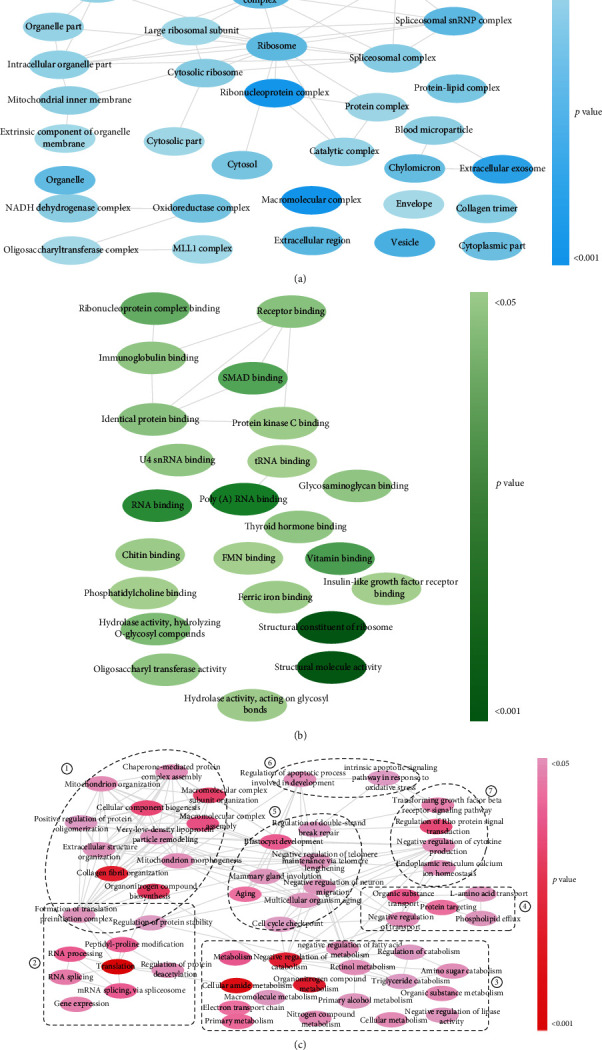
GO enrichment analysis of differentially expressed proteins. Clusters of GO terms (*p* < 0.05) for (a) cellular component, (b) molecular function, and (c) biological process were visualized using ReviGO. Node color intensity is scaled according to the *p* value.

To identify the different regulatory networks in NRP and LRP endometria, we next conducted GO annotation and enrichment analysis based on DEPs. A total of 181 significantly enriched GO terms (*p* < 0.05) were identified, including 55 cellular component terms, 23 molecular function terms, and 103 biological process terms. Then, ReviGO was used to process significantly enriched GO terms, and Cytoscape was used to present the grouped GO terms (Figure 2). As shown in cellular component terms (Figure 2(a)), DEPs were mainly located in intracellular organelle parts and membranes, spliceosomes, and electron transport chain complexes. Based on enriched molecular function terms, we found that DEPs were mainly related to binding functions and enzyme activities. Furthermore, 6 clusters of biological process terms (Figure 2(c)), including the cluster related to organization, assembling, and remodeling (①), the cluster related to genetic information processing (②), the cluster related to signaling and transduction (③), the cluster related to regulation (④), the cluster related to substance metabolism (⑤), and the cluster related to transportation (⑥), were enriched.

Of note, “collagen fibril organization,” “translation,” “negative regulation of catabolism,” “cellular amide metabolism,” and “organonitrogen compound metabolism” were the 4 most significantly enriched biological process terms, indicating the important role of organization/remodeling, translation, and substance metabolism in regulating endometrial function. More specifically, organization/remodeling-related DEPs are involved in terms of “collagen fibril organization,” “extracellular fibril organization,” “extracellular matrix structural constituent,” “extracellular structure organization,” “basement membrane disassembly,” and “vesicle organization” in LRP sows. Substance metabolism-related terms, such as nitrogenous substances metabolism (L-amino acid transportation, nitrogenous compound metabolism), lipid metabolism (“triglyceride catabolism,” “negative regulation of lipase activity,” “phospholipid efflux,” and “negative regulation of fatty acid metabolic process”), and retinol metabolism, were found in LRP proliferative endometria (Figure 2 and Figure S3).

### 3.3. KEGG Analysis of DEPs

**Figure 3:**
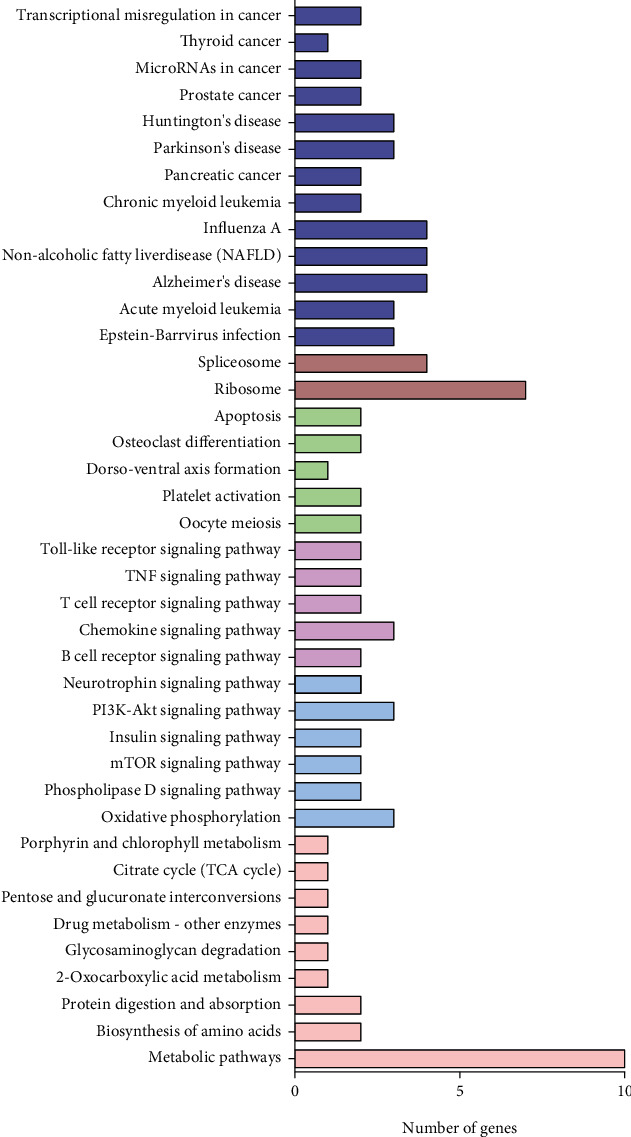
Cluster of top 40 enriched KEGG pathways based on differentially expressed proteins. Six clusters were enriched, which included (a) metabolic pathways, (b) regulatory functions, (c) immunological modulation, (d) reproduction and development, (e) genetic information processing, and (f) human diseases.

To depict pathways that regulate NRP and LRP endometrial function, KEGG enrichment analysis was performed. The top 40 enriched pathways are shown in Figure 3. These pathways were mainly related to metabolic pathways (a), regulatory functions (b), immunological modulation (c), reproduction and development (d), genetic information processing (e), and human diseases (f). Of note, we found that “biosynthesis of amino acids” and “protein digestion and absorption,” “oxidative phosphorylation,” and “citrate cycle (TCA cycle)” were significantly enriched. Interestingly, pathways related to metabolic regulation such as the “PI3K-AKT signaling pathway,” “insulin signaling pathway,” and “mTOR signaling pathway,” which are associated with organic insulin sensitivity [28], were also enriched. In addition, the “toll-like receptor signaling pathway,” “chemokine signaling pathway,” and “B cell receptor signaling pathway” were enriched in the immune regulation cluster.

### 3.4. Insulin Sensitivity Was Compromised in LRP Sows

**Figure 4:**
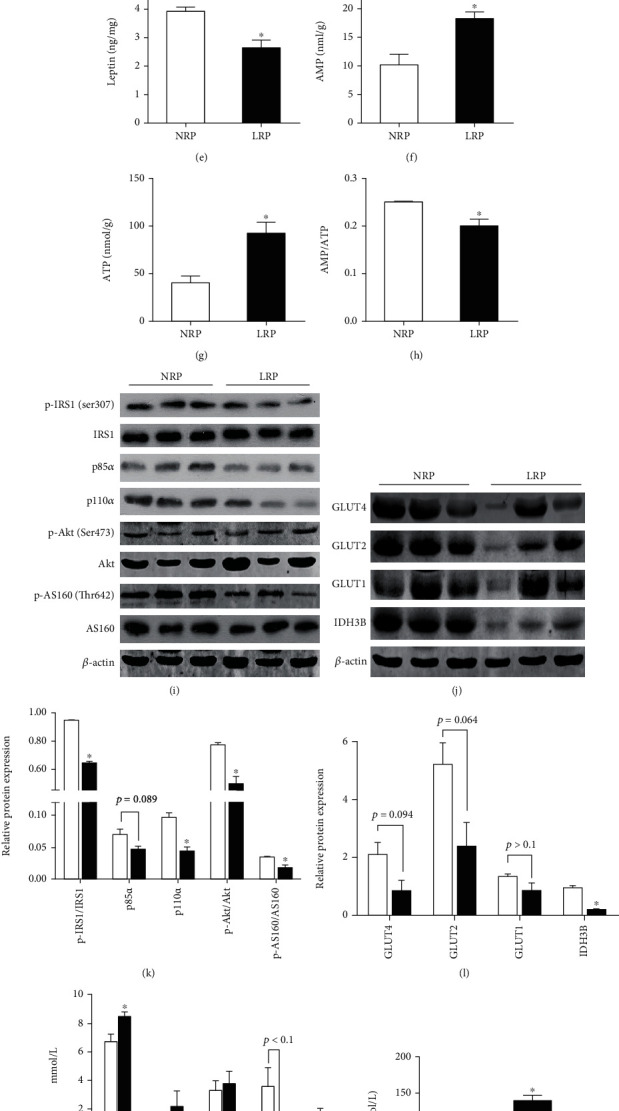
Serum and endometrial indicators implied a compromised insulin sensitivity in the endometria of LRP sows, compared with NRP sows. Levels of (a) TG, (b) malonyl-CoA, (c) DAG, (d) ceramide, (e) leptin, (f) AMP, (g) ATP, and (h) AMP/ATP in endometrial tissues. Representative blot (i) and quantification (k) of protein abundance of IRS1, p-IRS1^Ser307^, p85*α*, p110*α*, Akt, p-Akt^Ser473^, AS160, and p*-*AS160^Thr462^, and representative blot (j) and quantification (l) of protein abundance of GLUT4, GLUT2, GLUT1, and IDH3B in the endometria. Serum levels of (m) glucose, TG, CHOL, HDL-C, LDL-C, and (n) insulin. (o) HOMA-IR scores were used to reflect insulin sensitivity. *n* = 3. Data are expressed as mean ± SEM. Student's *t* test was used for statistical analysis. ^∗^*p* < 0.05. TG: triglyceride; CHOL: cholesterol; HDL-C: high-density lipoprotein cholesterol; LDL-C: low-density lipoprotein cholesterol; TG: triglyceride; DAG: diacylglycerol; NRP: normal reproductive performance; LRP: low reproductive performance.

As indicated above, disrupted lipid and protein metabolism and perturbed insulin signal were present in the endometria of LRP sows evidenced by GO and KEGG enrichment analysis. Given that endometrium is a peripheral tissue responsive to insulin, we postulated there might be impaired insulin sensitivity in the endometria of LRP sows. Therefore, lipid metabolites and insulin signaling were determined in the endometria of NRP and LRP sows. Levels of malonyl-CoA, diacylglycerol (DAG), and ceramide were higher (*p* < 0.05) (Figures 4(b)–4(d)), while leptin and AMP/ATP levels were lower (*p* < 0.05) (Figures 4(e)–4(h)) in the endometria of LRP sows compared with those of NRP sows, indicating the occurrence of abnormal lipid accumulation and oxidative phosphorylation in the endometrium of LRP sows. Importantly, Ser phosphorylation of IRS-1 was diminished in the LRP group (*p* < 0.05). The p110*α* protein level was also significantly reduced in the LRP group, while the p85*α* protein level tended to be decreased (*p* = 0.089) compared with the NRP group. Consistently, the phosphorylation of Akt and AS160 was decreased in the endometria of LRP sows (*p* < 0.05) (Figure 4(i)). We also found that protein expression levels of GLUT4 (*p* = 0.094) and GLUT2 (*p* = 0.064) tended to be decreased in LRP endometria compared with NRP counterparts, although the GLUT1 protein level was not altered in the endometria of sows with different reproductive performance (Figure 4(j)). In addition, the IDH3B protein level was lower in the endometria of LRP sows compared with NRP (*p* < 0.05, Figure 4(j)). Collectively, these data revealed that local insulin sensitivity was impaired in LRP endometria compared with that in NRP endometria.

Furtherly, we assessed systematic alteration of insulin sensitivity of NRP and LRP sows. As shown in Figure 4, concentrations of glucose and insulin were significantly increased in the serum of LRP sows (*p* < 0.05), while no changes were observed in TG, CHOL, and LDL-C (*p* > 0.05). Meanwhile, the level of HDL-C tended to be decreased in the LRP group (*p* < 0.1, Figure 4(m)). Noteworthy, the HOMA-IR score was significantly increased in LRP sows, indicating that systematic insulin sensitivity was impaired in LRP sows (Figure 4(o)).

### 3.5. Uterine Insulin Resistance Beginning before Pregnancy Impacted Endocrine Status and Compromised Implantation during Pregnancy

**Figure 5:**
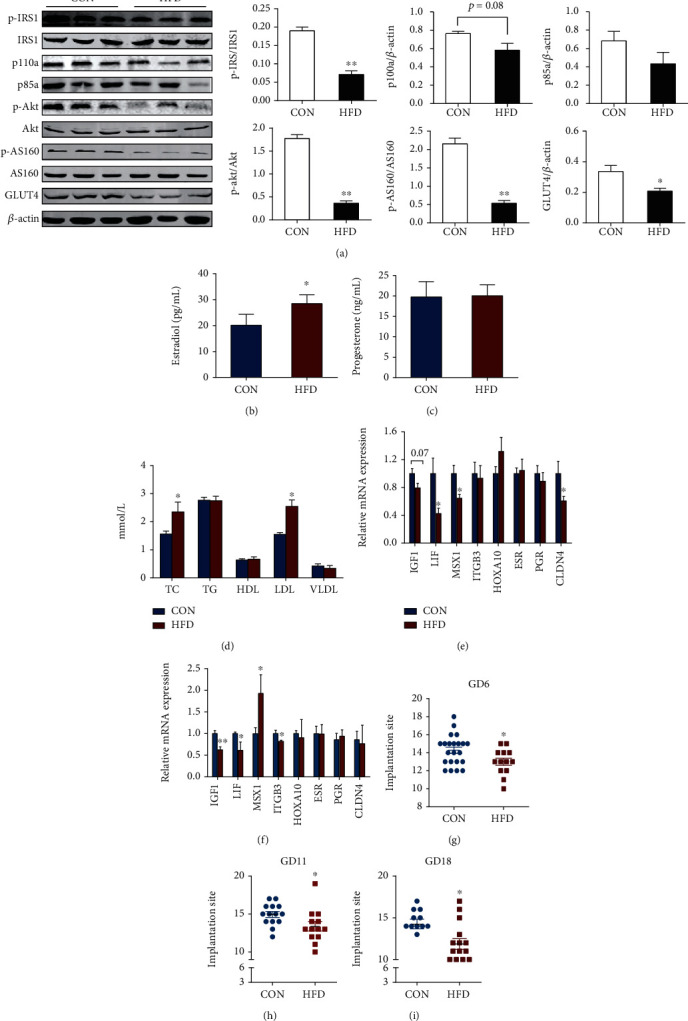
High-fat-induced-uterine insulin resistance beginning before pregnancy impaired endocrine status and embryo implantation in mice. Female mice of 8-week-age were fed with a CON diet or HFD (D12492 experimental diet) for 16 weeks and then mated with normal male mice. Day 1 of pregnancy was considered as the day when vaginal plug was observed. Uterine tissues and serum were collected at indicated time. (a) Representative blot and quantification of protein abundance of IRS1, p-IRS1^Ser307^, p85*α*, p110*α*, Akt, p-Akt^Ser473^, AS160, and p-AS160^Thr462^ in the uterus on day 5 of pregnancy. Levels of estradiol (b) and progesterone (c) and serum lipid changes (d) were determined on day 5 of pregnancy. Uterine receptivity-related gene changes on days 4 (e) and 5 (f) of pregnancy and the number of implantation site on days 6 (g), 11 (h), and 18 (i) of pregnancy were also recorded. Data are mean ± SEM values (*n* = 6 for (b–f), *n* = 14‐20 for (g)). Student's *t* test was used for statistical analysis. ^∗^*p* < 0.05 and ^∗∗^*p* < 0.01. CON: control; HFD: high-fat diet.

Insulin resistance during pregnancy links to adverse reproductive outcomes [29–31]. For example, diet-induced obesity and insulin resistance potentially impair endometrial stromal cell decidualization in mice in early stage pregnancy [31], insulin resistance during postimplantation period impairs mice uterine morphology, decidualization, and placentation processes [29], and overfeeding prior to parturition in dairy cows impaired insulin sensitivity and that negatively affected fertility [30]. Based on these observations, we speculated that uterine insulin resistance before pregnancy would negatively impact uterine functions and lead to adverse pregnant performance. To verify this hypothesis and further explore the possible mechanisms, an insulin-resistant mouse model was established by continuously feeding mice with a normal diet or HFD for 16~20 weeks beginning at 8 weeks of age. As expected, female mice that consumed the HFD, but not the control diet, developed glucose intolerance, insulin resistance (Figure S4), and alterations in insulin signaling (Figure S5A) before pregnancy. In addition, the aberrant higher glucose level continued with advancing gestation (Figure S5B) in the HFD group. Disruption of insulin signaling was confirmed in uteri during the peri-implantation period (gestational day 5). Briefly, protein expressions pIRS1, pAkt, and pAS160 were significantly decreased (*p* < 0.05), and p110*α* tended to be decreased (0.05 < *p* < 0.1) in uteri of mice fed HFD (Figure 5(a)). Importantly, GLUT4 protein expression was significantly depressed in the HFD group (Figure 5(a)), implying aberrant glucose metabolism in the uterus of HFD mice.

**Figure 6:**
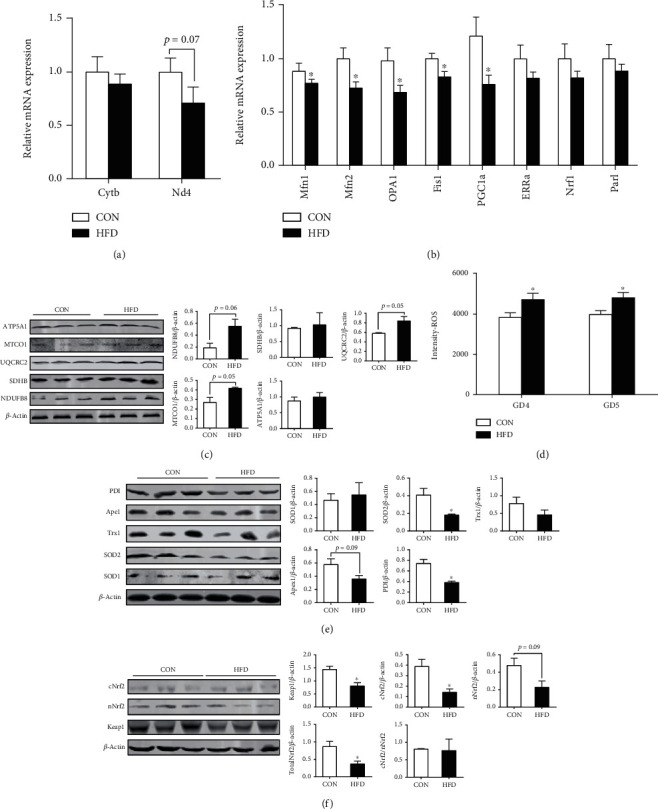
High-fat-induced-uterine insulin resistance beginning before pregnancy altered uterine mitochondrial function, leading to oxidative stress during the peri-implantation period in mice. Female mice fed with a CON diet or HFD for 16 weeks were mated with normal male mice. The day when vaginal plug was observed was considered as day 1 of pregnancy. Uterine samples were collected on day 5 of pregnancy. Uterine mtDNA copy number (a, *n* = 6), mitochondrial fission, and fusion genes (b, *n* = 6) were evaluated by qPCR. OXPHOS complex protein expressions were determined by western blotting with quantitation (c, *n* = 3). (d) Uterine ROS levels were determined using the DCFA-DA kit (*n* = 6). (e, f) Proteins involved in oxidative response (e) and defense (f) signaling pathway were determined by western blotting with quantitation. Student's *t* test was used for statistical analysis. ^∗^*p* < 0.05 and ^∗∗^*p* < 0.01. CON: control; HFD: high-fat diet.

Next, we investigated the reproductive parameters under uterine state of insulin resistance. We found that serum concentrations of estradiol increased (*p* < 0.05) in the HFD group compared with control-fed mice, but not the progesterone level (Figures 5(b) and 6(c)). Serum total cholesterol and LDL levels increased in the HFD group (*p* < 0.05, Figure 5(d)). Moreover, expression levels of genes related to implantation were analyzed, and mRNA expression levels of *Lif*, *Msx1*, and *Cldn4* were decreased (*p* < 0.05), and mRNA expression levels of *Ifg1* tended to be decreased (*p* = 0.07) in uteri of the HFD group on day 4 of the pregnancy (Figure 5(e)) compared with controls. *Igf1*, *Lif*, and *Itgb3* mRNA expression levels were decreased (*p* < 0.05), and the *Msx1* mRNA expression level was increased (*p* < 0.05) in the uterus of the HFD group on day 5 of the pregnancy (Figure 5(f)). Additionally, diminished implantation sites on day 6 and fetal numbers on days 11 and 18 of the pregnancy were observed (*p* < 0.05, Figures 5(g)–5(i)) in the HFD group compared with the control. Collectively, these data suggested that uterine insulin resistance impacted uterine receptivity, leading to reduced reproductive performance.

### 3.6. Mitochondrial Dysfunction-Induced Oxidative Stress in the Uterus with Insulin Resistance during the Peri-Implantation Period

Mitochondrial dysfunction and endoplasmic reticulum stress are features of peripheral insulin resistance and type 2 diabetes at the molecular, cellular, and organismal levels and might be the underlying cause of disturbed tissue function and homeostasis [29, 32, 33]. Here, we found that uterine mtDNA copy number tended to be decreased in HFD uteri (*p* = 0.07) compared with controls at gestational day 5 (GD5), indicated by decreased *Nd4* mRNA expression (Figure 6(a)). In addition, mRNA expression levels of genes involved in mitochondrial fusion (*Mfn1*, *Mfn2*, and *Opa1*), fission (*Fis1*) and biogenesis (*PGC1a*, *Erra*, and *Nrf1*), remodeling (*Parl*), and endoplasmic reticulum stress status (*Hspa5*, *Dd1t3*, *Hsp90b1*, *Erp44*, *Pdia3*, *Pdia4*, *Atfb*, *Eif2ak2*, *Bax*, and *Caspase12*) were estimated in the uterus at GD5 (Figure 6(b)). We found that mitochondrial fusion and biogenesis were dysregulated, indicated by decreased mRNA expression of *PGC1α*, *OPA1*, *Mfn1*, and *Mfn2* genes (*p* < 0.05, Figure 6(b)), during the peri-implantation period in HFD mouse uteri. By contrast, mRNA levels of genes related to endoplasmic reticulum stress were not changed (Figure S6).

Considering the dysfunction in mitochondria as indicated above, OXPHOS complex abundance, which is responsible for mitochondrial ROS production, was determined in uteri at GD5. As is shown in Figure 6(c), protein levels of three key components (ATP5A1, UQCRC2, and NDUFB8) of OXPHOS complexes tended to be increased in the HFD group (*p* = 0.05 ~ 0.06), which was consistent with proteomic data of sows which described that “mitochondrial organization,” “mitochondrial morphogenesis,” and “mitochondrial inner membrane” terms were dysregulated and respiratory chain complexes (NDUFA5, NUDFV2, and NDUFS5) were significantly increased in the endometria of LRP sow. As a result, uterine ROS production was increased in the HFD group at both GD4 (*p* < 0.05) and GD5 (*p* < 0.05) (Figure 6(d)). We further investigated the abundance of proteins involved in oxidative stress defense and response pathways in uteri at GD5. The expression of SOD2 and PDI deceased (*p* < 0.05), while Apex1 tended to be decreased (*p* = 0.09) in the HFD group compared with controls. In addition, the expression of Keap1, cNrf2, and total Nrf2 decreased (*p* < 0.05), while nNrf2 tended to be decreased (*p* = 0.09) in the HFD group (Figures 6(e) and 6(f)). Taken together, these data suggested that uterine mitochondrial dysfunction under insulin resistance differentially modulated the oxidative stress response of the uterus during the peri-implantation period.

### 3.7. ROS Overproduction Impaired Uterine-Embryo Implantation *In Vitro*

**Figure 7:**
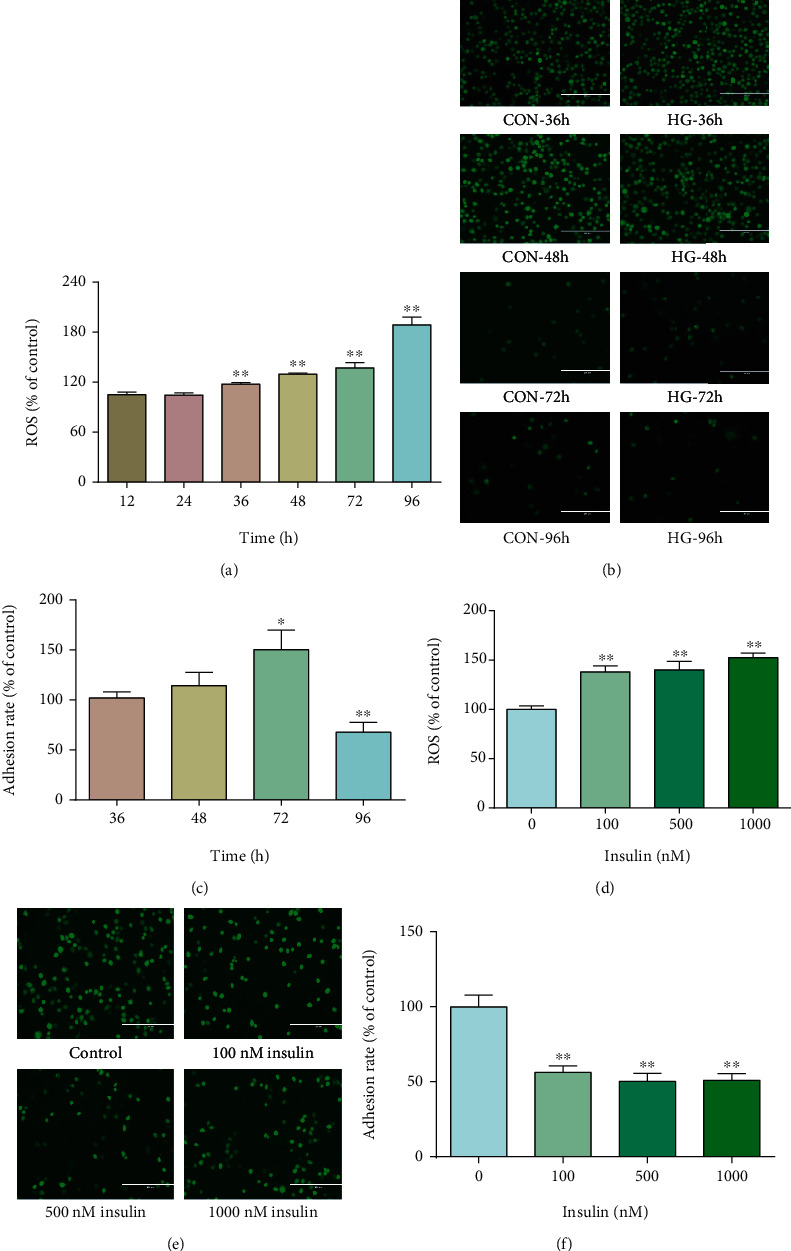
Oxidative stress impaired uterine receptivity *in vitro*. High glucose (41.1 mM) (a) or high insulin (100~1000 nM) (d) was used to induce oxidative stress in Ishikawa cells. (a, d) Cellular ROS levels were determined at the indicated time using the DCFA-DA kit. (b, e) JAR cells were stained with CellTracker™ Green CMFDA for 1 h before the adhesion assay and then plated onto treated Ishikawa cells for 1 h. Representative image of attached JAR cells to the Ishikawa cells after different treatment. (c, f) Adhesion rates were calculated. All experiments were performed at least three times. Data are mean ± SEM values. Student's *t* test was used for statistical analysis. ^∗^*p* < 0.05 and ^∗∗^*p* < 0.01. CON: control; HG: high glucose.

Cellular ROS could directly affect biological functions and signaling pathways. We hypothesized that cellular oxidative damage caused by ROS exposure might underly uterine dysfunction at implantation. Therefore, oxidative stress models were established by high glucose or high insulin treatment in Ishikawa cells to investigate the effect of elevated ROS production on embryo implantation *in vitro* (Figure 6). Ishikawa monolayer cells were pretreated with high glucose (41.1 mM) or high insulin (100, 500, or 1000 nM) for indicated time and then cocultured with CMFDA-labeled JAR cells. We found that ROS production was increased after 36, 48, 72, and 96 h of high glucose treatment (*p* < 0.05, Figure 7(a)). *In vitro* implantation indicated by an adhesion rate was increased for 72 h (*p* < 0.05), while significantly decreased for 96 h (*p* < 0.05) with high glucose treatment (Figures 7(b) and 7(c)). Similarly, the ROS level was increased by 100, 500, and 1,000 nM insulin treatment for 72 h (*p* < 0.05, Figure 7(d)). The adhesion rate was decreased by high insulin treatment for 72 h (*p* < 0.05, Figures 7(e) and 7(f)). Collectively, these results indicated that overproduction of ROS impaired uterine implantation.

## 4. Discussion

Implantation loss contributes greatly to the pregnancy loss for humans and mammalian animals [9, 34]. To dissect the underlying mechanism of proliferative endometrial defects in implantation loss, a proteomic analysis of endometrium from NRP and LRP sows was conducted in this study. GO and KEGG enrichment analysis revealed that DEPs were mainly involved in remodeling, immunological modulation, substance metabolism, and insulin signaling.

The endometria in the early proliferative phase are featured by appropriate tissue remodeling, angiogenesis, and modulation of immune function. During this period, the endometria are characterized by marks of the onset of expression of genes required for endometrial receptivity and a dampening of estrogen responsiveness [35]. The observation of impaired immunological modulation endometrial remodeling found in proliferative endometria of LRP sows, therefore, suggested that remodeling and immunological modulation defects in LRP proliferative endometrium might contribute to compromised endometrial receptivity and subsequent implantation loss in pregnancy.

In addition, pregnancy involves physiological changes and metabolic adaptations week by week. Amino acid requirement sharply increases during the peri-implantation period [36–38]. Accordingly, amino acids accumulate in the endometrium in response to progesterone [39]. Previous practices have demonstrated that dietary supplementation of amino acids, like arginine, leucine, or methionine, during pregnancy improves implantation by simulation of the PI3K/PKB/mTOR/NO signaling pathway, mTOR pathway [40, 41], and SAMTOR/mTORC1/S6K1/CAD pathway [42]. Besides, retinol metabolism and relevant signaling molecules, including binding proteins (CRBPs), synthesizing enzymes (Aldh1a1, Aldh1a2, and CRBP1), catabolizing enzymes (Cyp26a1), and receptors (RAR and RXR), are all expressed in the uterus [43, 44], which lay the foundation of retinol's involvement in endometrial development and maintenance, stromal decidualization, and blastocyst implantation [45]. Consist with this, vitamin A deficiency impacts histological and histochemical properties of the endometrium [46] and decidualization process [47]. However, little is known about amino acid or retinol metabolism related to endometrial functions during the proliferative phase. Based on the observations of abnormalities of L-amino acid transportation, nitrogenous compound metabolism, and retinol metabolism found in the endometria of LRP sow, we deduced that amino acid and retinol metabolism might regulate in endometrial functions during the proliferative phase.

What is more, high and low fertility endometria display discrepancies in the lipid metabolism during middle and late luteal phases, indicating that lipid metabolism is involved in the luteal phase endometrial functionality [11, 12]. Our group has proved that maternal short- and medium-chain fatty acid supply during early pregnancy enhances uterine phospholipid metabolism, leading to improved embryo survival [48]. Combined with the altered lipid metabolism observed in LRP proliferative endometria, it is deducible that lipid metabolism not only in the pregnancy period but also before pregnancy regulates the endometrium function for optimal pregnancy. Besides, long-chain acyl CoAs, DAG, and ceramides activate a host of serine kinases which negatively regulate insulin action [49]. Ceramide inhibits insulin action through reducing phosphorylated Akt protein expression, while DAG increases PKC-*θ* action which impairs insulin function via phosphorylation of IRS-1 [50]. Therefore, together with these studies, our observations of elevated malonyl-CoA, DAG, and ceramide in LRP endometria not only confirmed dysfunctional lipid metabolism but also further indicated a compromised uterine insulin sensitivity in LRP sows. The IRS1/PI3K/Akt pathway is a cascade of central signaling that mediates insulin's functions in glucose homeostasis in the body [51, 52]. Dysfunction of the IRS1/PI3K/Akt pathway responses to the abnormal insulin sensitivity. Here, we found alterations in the insulin signaling pathway, PI3K-Akt signaling pathway, and its downstream mTOR signaling pathway which were enriched in proteomic analysis and suppressed IRS1-PI3K-Akt signaling in LRP endometrium, as well as increased HOMA-IR index in LRP sows. Therefore, we concluded that uterine insulin sensitivity was impaired in LRP sows, which implied that proliferative endometrial defects in insulin sensitivity may potentially be one underlying mechanism of implantation loss. This thought is supported by that impaired glucose metabolism was observed in nonimplantative IVF cycle using proteomic analysis of endometrial fluid [53].

Insulin resistance during pregnancy links to adverse reproductive outcomes. During the postimplantation period, uterine hyperandrogenism and insulin resistance alter uterine morphology and impair decidualization and placentation processes [29]. Prior to parturition, overfeeding that induced impaired insulin sensitivity negatively affected fertility [30]. Here, our finding is that impaired uterine insulin sensitivity in the endometrium proliferative period was associated with low litter size and that uterine insulin resistance beginning before pregnancy lowers implantation sites and fetal number which broaden our understanding on the importance of appropriate insulin sensitivity before pregnancy.

Mitochondrial dysfunction and endoplasmic reticulum stress are features of peripheral insulin resistance and type 2 diabetes at the molecular, cellular, and organismal levels and might be the underlying cause of disturbed tissue function and homeostasis [29, 32, 33]. It is reported that hyperandrogenism and insulin resistance induce gravid uterine defects in association with mitochondrial dysfunction and aberrant reactive oxygen species production [29]. Therefore, together with the evidences that “mitochondrial organization,” “mitochondrion morphogenesis,” and “mitochondrial inner membrane” terms were dysregulated in the proliferative endometrium of LRP sows and that uterine insulin resistance beginning before pregnancy induced mitochondrial dysfunction (evidenced by decreased *Mfn2*, *Opa1*, and *Fis1*) during the implantation window in mouse uteri from the HFD group, we concluded that mitochondrial dysfunction, but not the endoplasmic reticulum stress, underlaid uterine insulin resistance-induced impaired reproductive performance.

Mitochondrial dysfunction will lead to oxidative stress. Agarwal et al. have reported the development of spontaneous and recurrent miscarriage associated with ROS-induced oxidative stress [54]. Therefore, our evidence that ROS production was increased in HFD mouse uteri implied that mitochondrial dysfunction-induced ROS overproduction was the underlying mechanism of insulin resistance-induced pregnancy loss. In support with this, we demonstrated that the increased ROS level decreased implantation rate *in vitro*. In addition, in consistent with the elevated ROS, oxidative stress response (SOD2 and PDI) and defense (Nrf2) pathways were dysregulated. SOD2 protein expression was decreased in our current observations of HFD mice. Nrf2, a critical transcriptional factor that regulates cellular redox homeostasis [55], is suppressed in the gravid uterus in PCOS-like pregnant rats [29], and mice specifically lacking Nrf2 increase oxidative stress and display impaired fetal development and placental function [56]. As the transcriptional product of Nrf2, decreased SOD2 protein expression might respond to the decreased Nrf2, and PDI decreased as a supplement. Collectively, these data demonstrated that deteriorated uterine function through mitochondrial dysfunction elevated oxidative stress under the insulin-resistant state, leading to compromised implantation sites and fetal numbers.

In conclusion, the current study demonstrated that uterine insulin resistance beginning before pregnancy resulted in implantation and fetal loss through mitochondrial dysfunction-induced oxidative stress. Nutritional strategies (especially those targeting lipid, nitrogenous, and retinol metabolism) could be applied before pregnancy to improve insulin sensitivity and enhance endometrial preparation, which optimize well reproductive outcomes.

## Data Availability

The data that support the findings of this study are available in the article or supplemental information. Other information if needed can be available from the corresponding author upon reasonable request.

## Abbreviations

AMP: Adenosine monophosphate
ATP: Adenosine triphosphate
CHOL: Cholesterol
DEPs: Differentially expressed proteins
FC: Fold change
FDR: False discovery rate
GD: Gestational day
GO: Gene ontology
GTT: Glucose tolerance test
HDL-C: High-density lipoprotein-cholesterol
HFD: High-fat diet
HOMA-IR: Homeostatic model assessment method
ITT: Insulin tolerance test
KEGG: Kyoto Encyclopedia of Genes and Genomes
LDL-C: Low-density lipoprotein-cholesterol
LRP: Low reproductive performance
NRP: Normal reproductive performance
PLS-DA: Partial least squares-discriminant analysis
TG: Triglyceride.

## Supplementary Materials

Supplementary Materials Supplemental Table 1: protein searching parameters used in sequence database searching and protein identification. Supplemental Table 2: antibodies for protein expression using western blot. Supplemental Table 3: gene primers used for quantitative RT-PCR analysis. Supplemental Figure 1: flow diagram of the label-free quantitative proteomic analysis of proliferative endometrial. Supplemental Figure 2: quality check for protein extraction for label-free proteomic analysis. Supplemental Figure 3: biological process terms of substance metabolism enriched by GO analysis based on the differentially expressed proteins. Supplemental Figure 4: insulin-resistant female mouse model was established by high-fat diet induction for 16~20 weeks from the age of 8 weeks. Supplemental Figure 5: uterine insulin resistance was established in high-fat-induced insulin-resistant mice. Supplemental Figure 6: endoplasmic reticulum stress-related gene expression at the mRNA level in the uterus during peri-implantation.
