# Supplementary material for: Uterine Insulin Sensitivity Defects Induced Embryo Implantation Loss Associated with Mitochondrial Dysfunction-Triggered Oxidative Stress
Source: Oxid Med Cell Longev. 2021 Apr 12;2021:6655685. doi: 10.1155/2021/6655685 (PMC8057892; doi:10.1155/2021/6655685)
Supplement: Supplementary Materials — Supplemental Table 1: protein searching parameters used in sequence database searching and protein identification. Supplemental Table 2: antibodies for protein expression using western blot. Supplemental Table 3: gene primers used for quantitative RT-PCR analysis. Supplemental Figure 1: flow diagram of the label-free quantitative proteomic analysis of proliferative endometrial. Supplemental Figure 2: quality check for protein extraction for label-free proteomic analysis. Supplemental Figure 3: biological process terms of substance metabolism enriched by GO analysis based on the differentially expressed proteins. Supplemental Figure 4: insulin-resistant female mouse model was established by high-fat diet induction for 16~20 weeks from the age of 8 weeks. Supplemental Figure 5: uterine insulin resistance was established in high-fat-induced insulin-resistant mice. Supplemental Figure 6: endoplasmic reticulum stress-related gene expression at the mRNA level in the uterus during peri-implantation. [file 6655685.f1.docx]

**Supporting Information**

**Supplemental materials and methods**

**Proteomic Sample preparation and Label-free proteomics analysis of endometrial tissues**

Proteomic samples were prepared using endometria from NRP and LRP sows were powdered in liquid nitrogen and lysed in a buffer containing 40 mM Tris–HCl, 7 M urea, 2 M thiourea, 4% w/v CHAPS, 1% DTT, 1 mM EDTA and 1× protease inhibitors cocktails (Thermo Fisher Scientific, Rockford, USA). Protein concentration was determined using the Bradford Protein Assay Kit (Thermo Fisher Scientific, Rockford, USA) according to the manufacturer’s instruction. Protein sample (200 μg) was mixed with 200 mM NH_4_HCO_3_ and then vigorously vortexed. Subsequently, the mixture was reduced by DTT with its final concentration of 100 mM. After incubated at 56°C for 1 h, the mixture was cooled to room temperature, added with IAA for final 55 mM IAA, and incubated for an additional 40 min at room temperature in darkness. Next, trypsin (5 μg) was added to the sample and then incubated at 37°C for 16 h. Finally, tryptic peptide mixtures were collected and kept at -80°C for further analysis.

Label-free analysis of peptides were achieved on an Eksigent 425 (AB SCIEX) LC system coupled with Q-Exactive mass spectrometer (Thermo Scientific, Bremen, Germany). Each sample (2 μL) was injected under the auto-injection program mode and pre-concentrated on a self-packed C18 trap column (3 μm, 0.1×20 mm, Thermo Scientific, Bremen, Germany). Separation was achieved using an analytical C18 column (5μm, 0.75×150 mm, Thermo Scientific, Bremen, Germany) at a flow rate of 300 μL/min. Mobile phase A was 0.1% formic acid in water and phase B was 0.1% formic acid in acetonitrile/H_2_O (80%׃20%, v/v). The gradient program went from 5% B up to 8% B in 5 min, from 8% B up to 15% B in 30 min, from 15% B up to 30% B in 25 min, from 30% B up to 45% B in 20 min, then increased to 80% B in 20 min. The gradient then held consistent for 10 min with 80% B, accompanied by a sharp decrease from 80% B to 5% B in 0.1 min, and then maintained the volume ratio of 5% B for 10 min. The total time of the curved gradient was 120 min. The resolution, automatic gain control (AGC) target value, maximum injection time and dynamic exclusion of the full-scan MS spectra were set at 70,000 at m/z 200, 3e6, 80 ms and 25.0 s, respectively. Higher energy collision-induced dissociation (HCD) fragmentation was performed for tandem data-dependent analysis for the 20 most intense signal ions selected per cycle. The normalized collision energy, resolution, AGC target value and maximum injection time were set at 30%, 17,500 at m/z 110, 3e6 and 45 ms, respectively. The isolation window and intensity threshold were set at 2.0 m/z and 2e5, respectively.

**Sequence database searching and protein identification**

Mass data were depicted by Mascot 2.2 and Proteome Discoverer software 2.0 against a Uniprot-pig.fasta database. Protein searching parameters were listed in **Supplemental Table 1**. Peptide mass tolerance and fragment mass tolerance were set as ±15 ppm and 20 mmu, respectively. The peptide confidence was high, and the peptide length was set > 6. The false discovery rate (FDR) was strictly limited to < 0.01. The NRP group was set as the control of the LRP group. Proteins with a fold change (FC) > 1.5 (or < 0.6667) and *p* < 0.05 were regarded as differentially expressed proteins (DEPs).

***In vitro* implantation model**

*In vitro* implantation was performed using Ishikawa and JAR cells as described previously with minor modification [1]. Ishikawa cells were grown in 96-well plates with glucose (11.1 and 41.1 mM) or insulin (0, 100, 500 and 1000 nM) for 12~96 h. JAR cells were stained with CellTracker™ Green CMFDA (Invitrogen, Carlsbad, CA) for 1 h before the cell adhesion assay was conducted. For uterine receptivity assay, the stained JAR cells (5000~10000 per well) were plated onto the treated Ishikawa cells and co-cultured for 1 h. Then the unattached JAR cells were removed gently by washing with PBS three times. Attached cells were pictured using a fluorescent microscope system (Olympus, Japan). Adhesion rates were calculated as percentage of attached JAR cells to total JAR cells. All experiments were performed at least three times.

**Supplemental results**

**Supplemental table 1. Protein searching parameters used in Sequence database searching and protein identification.**

| Item | Value |
| --- | --- |
| Mascot | 2.2 |
| ProteomeDiscverer version | 2.0 |
| Protein Database | Uniprot-pig.fasta |
| Cys alkylation | Iodoacetamide |
| Enzyme Name | Trypsin (Full) |
| Dynamic Modification | Oxidation (M), Acetyl (Protein N-Terminus) |
| Static Modification | Carbamidomethyl © |
| Max. Missed Cleavage Sites | 2 |
| Precursor Mass Tolerance | 10 ppm |
| Fragment Mass Tolerance | 0.02 Da |
| Validation based on | Q-value |

**Supplemental table 2. Antibodies for protein expression using western blot.**

| **Antibodies** | **Sources** | **Identifiers** | **Dilution ratios** |
| --- | --- | --- | --- |
| CSTB | Cusabio | CSB-PA00224A0Rb | 1:400 |
| ADAM10 | Cusabio | CSB-PA909378 | 1:500 |
| HSD11B1 | Cusabio | CSB-PA010763LA01HU | 1:400 |
| CTSS | ImmunoWay | YN2060 | 1:500 |
| SLC25A22 | Proteintech | 25402-1-AP | 1:500 |
| IRS1 | Cell Signaling Technology | #2382 | 1:1000 |
| phospho-IRS1 (Ser307) | Cell Signaling Technology | #2381 | 1:500 |
| PI3 Kinase p85 | Cell Signaling Technology | #4292 | 1:1000 |
| PI3 Kinase p110α | Cell Signaling Technology | #4255 | 1:1000 |
| phospho-AKT (Ser473) | Cell Signaling Technology | #9271 | 1:500 |
| AKT | Cell Signaling Technology | #9271 | 1:1000 |
| phospho-AS160 (Thr642) | Cell Signaling Technology | #8881 | 1:500 |
| AS160 | Cell Signaling Technology | #2670 | 1:1000 |
| GLUT4 | Cell Signaling Technology | #2213 | 1:1000 |
| GLUT2 | Bioss | bs-0351R | 1:500 |
| GLUT1 | Bioss | bs-4855R | 1:500 |
| IDH3B | Abcam | Ab112544 | 1:500 |
| ATP5A1 | Proteintech | 14676-1-AP | 1:1000 |
| MTCO1 | Abcam | ab203912 | 1:1000 |
| UQCRC2 | Proteintech | 14742-1-AP | 1:500 |
| SDHB | Proteintech | 10620-1-AP | 1:500 |
| NDUFB8 | Proteintech | 14794-1-AP | 1:500 |
| PDI | Proteintech | 11245-1-AP | 1:500 |
| Ape1 | Proteintech | 10203-1-AP | 1:500 |
| Trx1 | Proteintech | 14999-1-AP | 1:500 |
| SOD1 | Proteintech | 10269-1-AP | 1:500 |
| SOD2 | Proteintech | 24127-1-AP | 1:500 |
| Nrf2 | Proteintech | 16396-1-AP | 1:500 |
| Keap1 | Proteintech | 10503-2-AP | 1:500 |
| β-actin | Cell Signaling Technology | #4970 | 1:10000 |

**Supplemental table 3. Gene primers used for quantitative RT-PCR analysis.**

| Gene (mouse) | Forward primer (5'-3') | Reverse primer (5'-3') | Product size (bp) |
| --- | --- | --- | --- |
| *IGF1* | CACATCATGTCGTCTTCACACC | GGAAGCAACACTCATCCACAATG | 220 |
| *LIF* | GCCTCCAGGTCAAGCTCAAT | TTGTTGCACAGACGGCAAAG | 79 |
| *MSX1* | ACCACCTGGTCCCTTCTCTT | GCTGGGGACCACGGATAAAT | 208 |
| *ITGB2* | CCCCGATGTAACCTGAAGGAG | GAAGGGCAATCCTCTGAGGG | 166 |
| *HOXA10* | CCTGCCGCGAACTCCTTTT | GGCGCTTCATTACGCTTGC | 203 |
| *ESR1* | AATGCAAGAACGTTGTGCCC | TCTGCTTCCGGGGGTATGTA | 181 |
| *PGR* | TATGGCGTGCTTACCTGTGG | TGCCAGCCTGACAACACTT | 160 |
| *CLDN4* | AGCAAACGTCCACTGTCCTT | GGGGCGTAATGGCAAGAGTA | 80 |
| *Mfn1* | GGCAGGACAAGTAGTGGCAAGAG | CGACACTCAGGAAGCAGTTGGTT | 103 |
| *Mfn2* | AAGCAGATTACGGAGGAAGTGGAA | GAGCAGCGGTCAGACAGGTT | 101 |
| *OPA1* | AGGATGGTGCTCGTGGACTTG | ACAGGATGATGGCGTTAGGATTCT | 127 |
| *Fis1* | TTGAATATGCCTGGTGCCTGGTT | GCTGTTCCTCTTTGCTCCCTTTG | 105 |
| *PGC1a* | TCGCTGCTCTTGAGAATGGATATACTT | AATCGTCTGAGTTGGTATCTAGGTCTG | 126 |
| *ERRa* | GGCTTCTCCTCACTGTCACTGTCT | GCACCAGCACTTCCATCCACAC | 70 |
| *Nrf1* | CTGTGGCTGATGGAGAGGTGGAA | GATGCTTGCGTCGTCTGGATGG | 79 |
| *Parl* | CTTCTCATCTAGCATCGTGAACATCCT | GGTCCATATCTTCCTGTGGCAACTT | 123 |
| *Hspa5* | CCTTGTGTTTGACCTGGGTG | CCATGACCCGCTGATCAAAG | 131 |
| *Hsp90b1* | AGTCGGGAAGCAACAGAGAA | TCTCCATGTTGCCAGACCAT | 160 |
| *Ddit3* | TCACTACTCTTGACCCTGCG | GACTGGAATCTGGAGAGCGA | 102 |
| *Erp44* | GCCAGAGATAGGAGCAGAGG | GAGCCAATTTAGAGCCTGCC | 86 |
| *Pdia3* | AGTATGAAGGTGGCCGTGAA | GAGGTCCTCTTGTGCCTTCT | 119 |
| *Pdia4* | GTCTCTGGCTACCCGACTTT | GCCCAGACTGCTCAATCATG | 109 |
| *Bax* | CTGGAAGAAGATGGGCTGAG | CATTCCCACCCCTCCCAATA | 139 |
| *Eif2ak3* | TTGGCCACTTTGAACTTCGG | CGCCATGACCTTCCAATCAG | 182 |
| *Atf6* | TCAGCCTGGCCTACATTTCA | TATGGTGAAGGAAGCAGGCA | 168 |
| *Caspase12* | GGAAGGTAGGCAAGACTGGT | TGACTGGGAACTGCATGAGA | 227 |
| *Cytb* | ACAAAGCCACCTTGACCCGATTC | GAGGAAGAGGAGGTGAACGATTGC | 89 |
| *Nd4* | CGCCTACTCCTCAGTTAGCCA | TGATGTGAGGCCATGTGCGA | 112 |
| *β-actin* | GCAGGAGTACGATGAGTCCG | ACGCAGCTCAGTAACAGTCC | 74 |

**Supplemental Figure 1.**


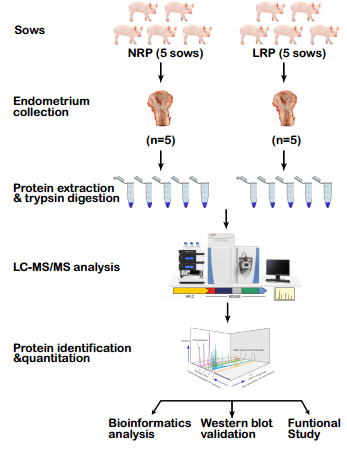


**Supplemental Figure 1. Flow diagram of the label-free quantitative proteomic analysis of proliferative endometrial.** Endometrial tissues were collected from 10 sows with different reproductive performance. Proteins were extracted and digested. The obtained peptides were analyzed by high-resolution LC−MS/MS and quantified with the label-free algorithm in Mascot 2.2 and Proteome Discoverer software 2.0. The differentially expressed proteins were further analyzed by GO, KEGG pathway enrichment and protein−protein interaction network analyses and followed by biological validation and functional study using western blot.

**Supplemental Figure 2.**


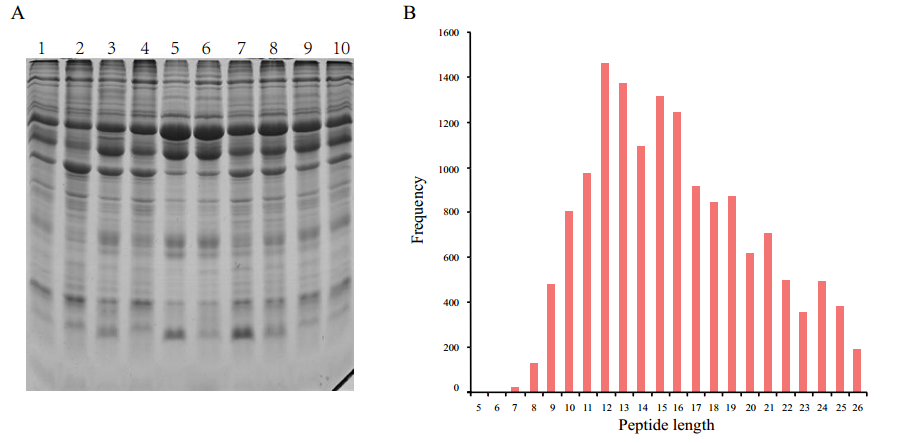


**Supplemental Figure 2. Quality check for protein extraction for label-free proteomic analysis.** (A) The same aliquot protein extract (50 μg per sample) was run on a 10% SDS-PAGE gel. Sample lanes 1, 2, 4, 7 and 9 represented protein extracted from endometrium with normal reproductive performance. The rest represented protein extracted from endometrium with low reproductive performance. (B) Distribution of peptide length. Tryptic peptides were analyzed by LC coupled with Q-Extractive mass spectrometry.

**Supplemental Figure 3.**

**Supplemental Figure 3. Biological process terms of substance metabolism enriched by GO analysis based on the differentially expressed proteins.**

**Supplemental Figure 4.**


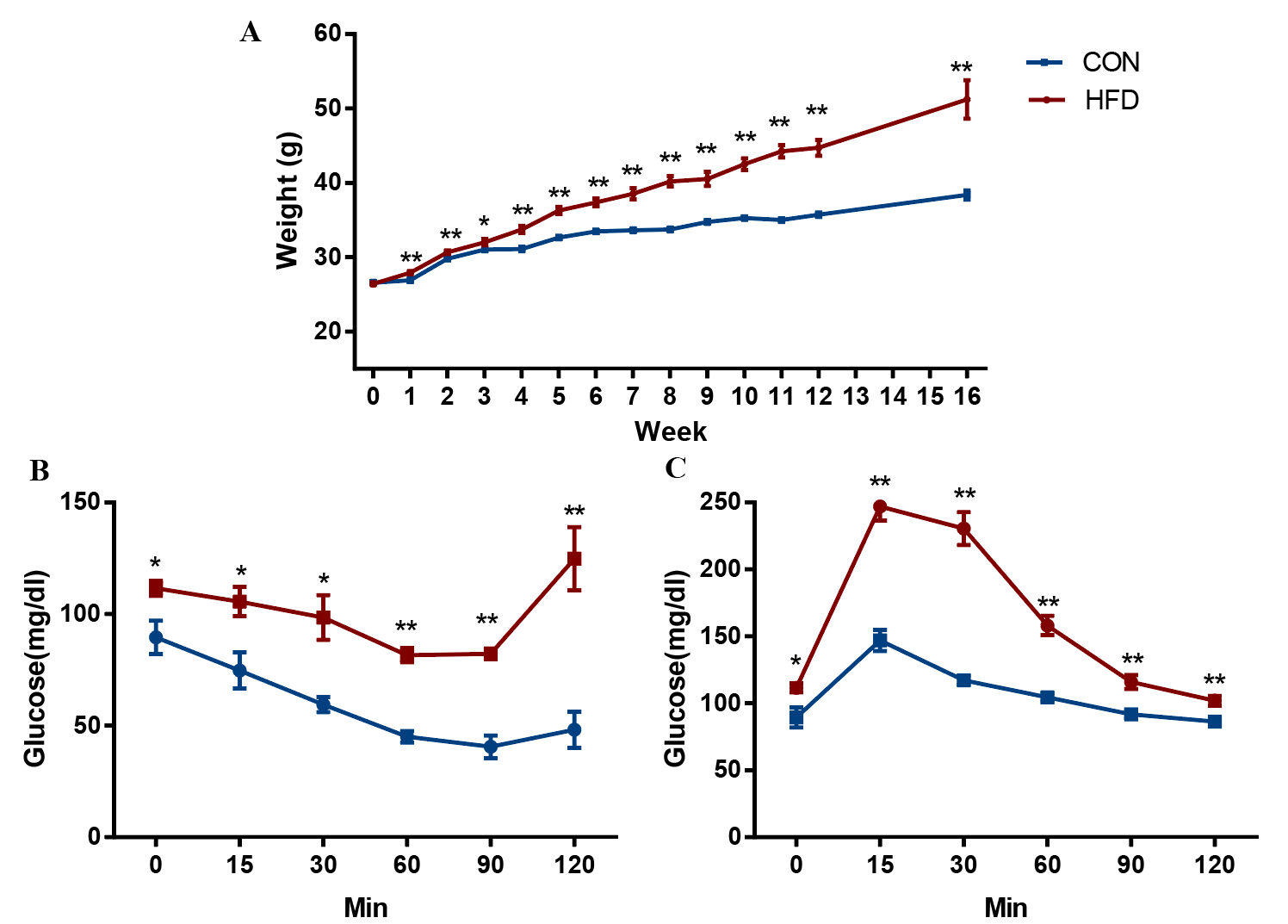


**Supplemental Figure 4. Insulin resistant female mouse model was established by high-fat diet induction for 16~20 weeks from the age of 8 weeks.**

To establish a diet-induced model of insulin resistance, female mice were continuously fed with a control diet or a high fat diet (HFD) for 16~20 weeks beginning at 8 weeks of age. As expected, female mice that received the HFD became obese, insulin tolerant and glucose tolerant, while mice that received the control diet did not. (A) weight change, (B) Insulin tolerance tests, (C) glucose tolerance tests. At least 6 mice were used in each group. Data are expressed as mean ± SD. Student’s *t* test was used for statistical analysis between the control and HFD group. **p* < 0.05, ***p* < 0.01.

**Supplemental Figure 5.**


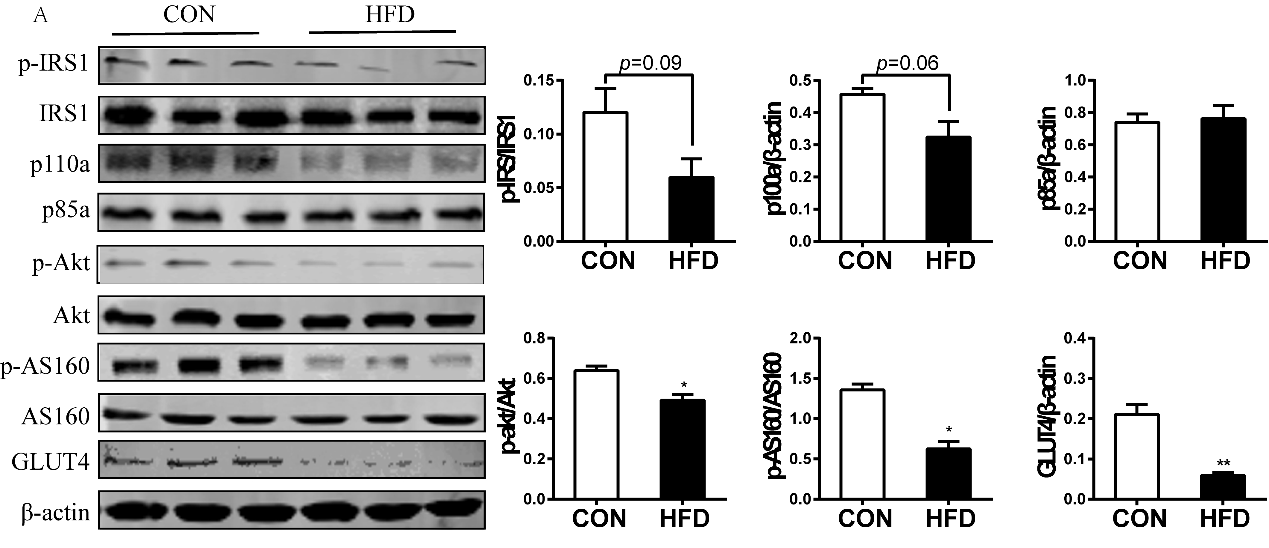

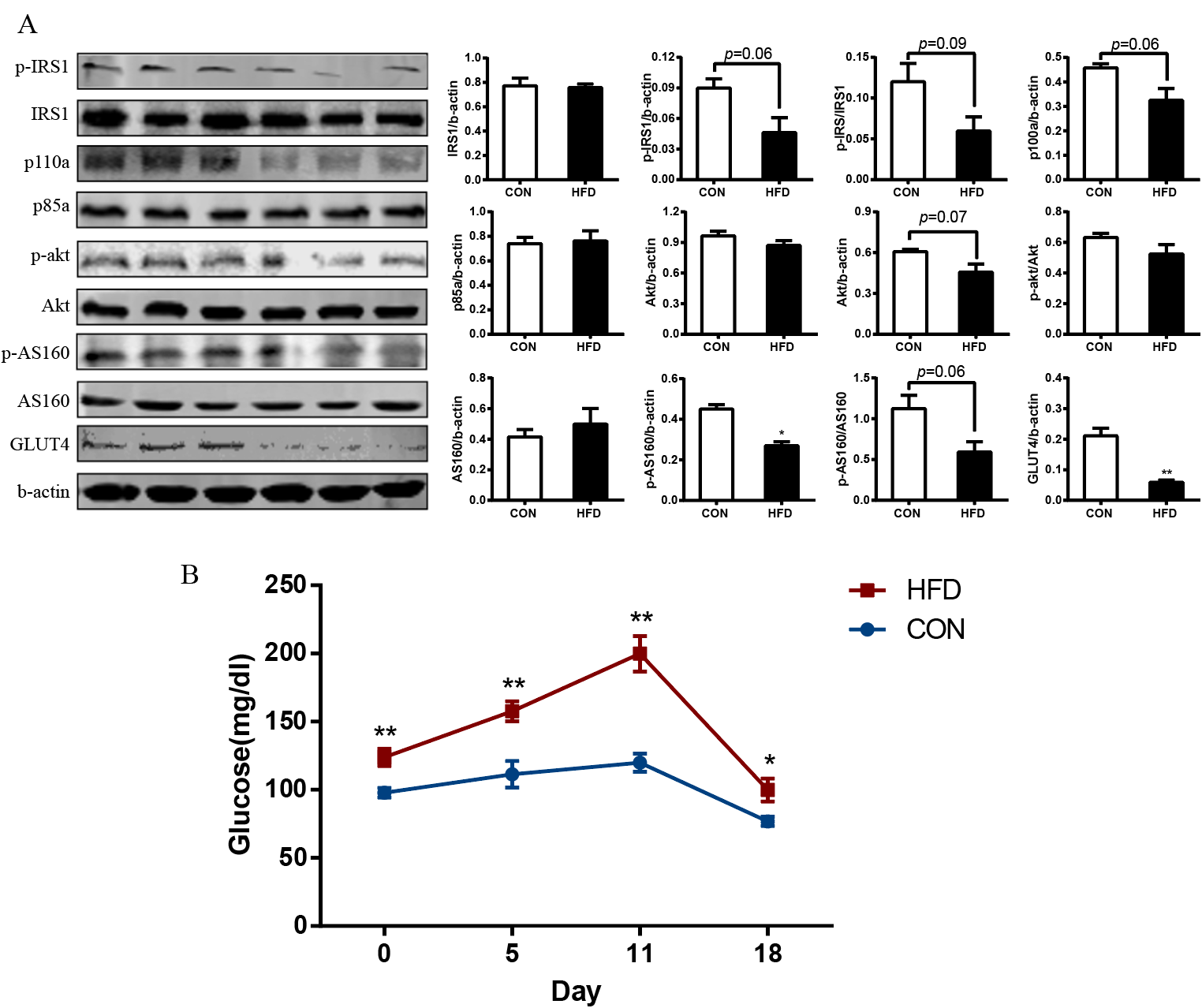


**Supplemental Figure 5. Uterine insulin resistance was established in high-fat induced insulin resistant mice.**

Female mice (8-week of age) were fed with a control (CON) or high fat (HFD) diet for 16~20 weeks. Uterine samples were collected. (A) High-fat induced insulin signaling molecule alteration in the uterus. (n = 3) (B) Aberrant high glucose level continued during the pregnancy period in high-fat-feeding mice. (n = 6). Data are expressed as mean ± SEM. Student’s *t* test was used for statistical analysis between the control and HFD group. **p* < 0.05, ***p* < 0.01.

**Supplemental Figure 6.**


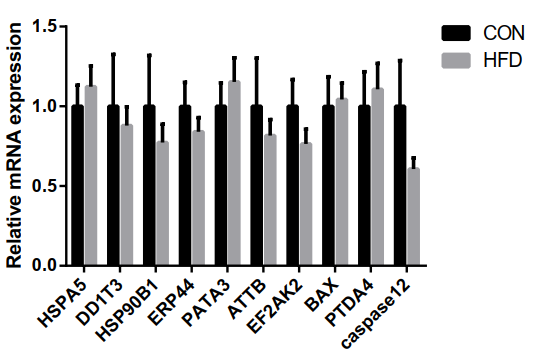


**Supplemental Figure 6. Endoplasmic reticulum stress-related gene expression at mRNA level in uterus during peri-implantation.** Female mice of 8-week-age were fed with a control (CON) or high fat (HFD) diet for 16~20 weeks, and then mated with normal male mice. Day 1 of pregnancy was considered as the day when vaginal plug was observed. Samples were collected on day 5 of pregnancy (n = 6). Data are expressed as mean ± SD. Student’s *t* test was used for statistical analysis between control and HFD group. **p* < 0.05.

References:

1. M. Yu, H. Qin, H. Wang, J. Liu, Q. Yan, J Cell Physiol. **2020,** 235:1076-1089.
